# Why be a refugee camp doctor: the challenges, rewards and medical education aspects

**DOI:** 10.5116/ijme.5985.c959

**Published:** 2017-08-18

**Authors:** Hanina Abi Nader, William Watfa

**Affiliations:** 1 Department of Medicine, Johns Hopkins University School of Medicine, Baltimore, USA; 2 Department of Plastic, Reconstructive and Hand Surgery, University Hospital of Lausanne (CHUV), Lausanne, Switzerland

**Keywords:** Refugee camp doctor, medical education aspects

## Introduction

Since the Syrian conflict erupted in March 2011, the United Nations High Commissioner for Refugees (UNHCR) recorded the number of Syrian refugees registered in Lebanon, as of December 2016, to have reached 1,011,366, making Lebanon a country with the highest refugee population per capita approaching 2:1.^1^ In addition, close to 27% of households among the displaced Syrian population count at least one member with a specific need such as a chronic disease or disability. More than 50% of the children are deeply traumatized.^2^ Most refugee camps struggle to access primary care services, and the lack of doctors willing to commit to that particular practice makes it even harder to provide camps with appropriate care.^3^ In response to those distinctive needs of refugees, some non-governmental organizations try to provide these camps with volunteer doctors that are recognized as “refugee camp doctors”.

Nowadays, most modern medical curricula are based on clinical experience that is learned in an almost “ideal setting”. At the hospital or the clinic, medical students and residents will take patients history, perform physical exams, order a workup or start treatment, while issues related to finance, gender, politics or even climate are rarely taken into consideration in their routine. However, when practicing as a refugee camp doctor, one discovers that those factors become part of the main elements that constitute medical practice. The experience gained in the field is somehow different from that offered by the traditional medical education. As a remedy, the adoption of a novel approach that promotes new medical skills is required. Hence, this perspective aims to describe the experience of both authors as volunteer camp doctors and to stress the added value to their medical curriculum.  It also identifies the skills that are needed to address the challengesencountered under precarious field conditions adequately.

### Health and humanitarian response

The authors (H.A.N. and W.W.) accepted the commitment as volunteer camp doctors.  Armed with insatiable curiosity, hands-on skills, they decided to enrich their experience in community medicine by exploring a new location that had neither a curriculum nor an advanced medical facility. While both completed medical school at the same time, H.A.N. had a one-year internship in internal medicine and an additional year of residency in paediatrics, and W.W. had completed his second post-graduate year in plastic and reconstructive surgery.

While their previous experience as residents in an underserved area had already exposed them to complex wound cases, challenging psychosocial issues and common paediatric viral cases, the health problems at the camp were by far more varied. No residency program could have prepared a doctor for the spectrum of issues encountered in the camp. 

### Life, health conditions and resources at the camp

The crowded refugee camp population was housed in worn out tents with torn roofs and rotten bottoms that would succumb to the first rain. Refugees were severely deprived, with no access to potable water, food or medical care. Ironically, the leaking tent roofs served as their only shower facilities. Joblessness created tensions, and ill feelings became a threat to civil order. Children walked barefoot on rough terrain, with light clothing that was not enough to protect their vulnerable skin from harsh climatic conditions. With its dense refugee population, deprivation, crowded living conditions, and scarcity of sanitary and washing facilities, the camp defied all the required basic public health prerequisites.

The medical team consisted of a volunteering pharmacist and the two medical doctors. “The clinic” was a tent shaded by a tree on sunny days. Patients were divided into groups, starting with those who presented more urgent symptoms such as fever or pain. The second group was offered a general examination. Most of the patients complained of viral illnesses, which required supportive treatment and appropriate follow-up. In an average clinic, the doctor would examine thirty patients with diagnoses ranging from burns to scabies. The medical team’s testing ability was restricted to a blood pressure cuff and a blood glucose monitor. Besides medical skills, the role of a camp doctor covered health education with an emphasis on child and maternal health, contraceptive use, preventive medicine and primary health care treatment with whatever limited medications that were available. An emergency kit was also available with at least one epinephrine auto-injector. When faced with a complex wound, debridement and suturing were performed using the limited material provided, with great vigilance needed to maintain a clean and sterile environment. Many patients presented with severe burn sequelae, and when possible was transferred to the Capital’s burn unit for specialised care. Antibiotics were scarce and restricted to cephalosporin. The task of delivering healthcare to men and women with 350 children, under the camp conditions referred to above, was not easy, especially when this had to be undertaken under a bushy tree to escape a leaking tent during an unexpected winter shower.

In the process, some other health issues were encountered. The crowded unsanitary living conditions were ideal for Giardiasis diarrheal outbreaks. Child labour was rampant among boys and girls, often exposed to carcinogens and systemic poisons such as insecticides. On the psychosocial front, the situation was not much more favourable as many individuals presented with psychological traumas including post-traumatic stress disorder. Reports of sexual assault including rape were common. Many times, when dealing with such patients one could only triage to a higher healthcare facility, but the likelihood of the patient getting there was meagre. 

### Lessons learnt as a medical volunteer

In addition to acquiring new medical skills, a medical volunteer is also taught humanism. Dedication, stamina, perseverance, creativity and compassion are required. Work meant ten long hours daily, not counting the three hours of round-trip driving to get to the camp. The medical team’s vehicle would be packed with donations in the form of basic children's needs such as diapers and powder milk. Thanks to its multilingual skills, the team can reach out to people of different cultures. However, despite language familiarity, a male doctor had to be cognizant of the sensitivity of examining female patients. Finally, when one clinic receives thirty patients per day, the experience also teaches time-management and efficiency.

The camp environment had no frills other than a hospitable and modest lunch invitation by dignified refugees who shared whatever nourishment they had on that particular day.  The refugees’ sincerity and their desire to “pay back” were touching. Other basic ingredients in accomplishing medical missions such as this one must include the volunteer's impartiality to religious and political affiliations of the target group, adaptability to work in a no-frill environment, and the acclimatization to work in a potentially volatile zone that is prone to the instantaneous eruption of armed struggle. Although the mission involved personal safety concerns, the humanitarian aspect surmounted all fears.

## Conclusions

Rural medical education is needed as a response to the problem of inaccessible health care. A volunteer camp doctor is taught much more than medicine: this clinical experience can be used to acquire new skills such as adaptability, improvisation, collaboration, endurance, and resilience. Medical education curricula must eventually include more emphasis on rural health medicine.

### Acknowledgments

The authors wish to thank Professor Nisha Chandra and Dr Shaker M. Eid for their valuable advice in the realization and presentation of the manuscript.

### Conflict of Interest

The authors declare that they have no conflict of interest.
